# Stomatal Morphology, Leaf Nutrients and Molecular Adaptations Are Associated With Scald Resistance in Barley

**DOI:** 10.1111/ppl.71117

**Published:** 2026-09-20

**Authors:** Usman Ijaz, Muhammad Atif Azeem, Sergey Shabala, Meixue Zhou, Chenchen Zhao

**Affiliations:** ^1^ Tasmanian Institute of Agriculture, University of Tasmania Launceston Tasmania Australia; ^2^ International Research Centre for Environmental Membrane Biology Foshan University Foshan China; ^3^ School of Biological Science University of Western Australia Crawley Australia

**Keywords:** antioxidants, barley, cytosolic Ca^2+^, scald, stomatal regulation

## Abstract

Scald, caused by *Rhynchosporium secalis*, can reduce grain yield by up to 40% in susceptible cultivars. To investigate physiological and molecular responses associated with scald resistance in barley, we compared a susceptible cultivar, Atlantis, with a resistant cultivar, Yangsimai 3, under uninoculated control and *R. secalis*‐inoculated conditions. Relative to the uninoculated control, Atlantis showed significant reductions in shoot and root length (20.6% and 22.9%), shoot and root dry weight (27.7% and 28.3%), SPAD value (30.9%) and chlorophyll fluorescence (10.8%), whereas Yangsimai 3 showed smaller changes. Under *R. secalis* inoculation, Yangsimai 3 showed lower stomatal conductance and smaller stomatal pore size, more stable K^+^ and Ca^2+^ contents, lower malondialdehyde (MDA) and hydrogen peroxide (H_2_O_2_) levels and higher superoxide dismutase (SOD, ~32%), peroxidase (POD, ~41%) and catalase (CAT, ~31%) activities, indicating stronger antioxidant capacity. qRT‐PCR analysis further showed generally enhanced gene expression in inoculated Yangsimai 3 involving Ca^2+^ signalling (*HvCAM1*, *HvCAM24* and *HvCDPK3*), K^+^ transport (*HvHAK1* and *HvGORK*), antioxidant defence (*HvCDS1, HvSOD2* and *HvCAT1*), phytohormone signalling (*HvAOC, HvLOX2* and *HvJIP60*), stomatal regulation (*HvOST1* and *HvSLAC1*) and MAPK‐mediated immune signalling (*HvMPK3* and *HvMPK14*), whereas reactive oxygen species (ROS) production was more strongly induced in Atlantis. These results suggest that scald resistance in Yangsimai 3 is associated with stomatal regulation, ion homeostasis, antioxidant defence and defence‐related gene expression.

## 1 Introduction

Barley (*Hordeum Vulgare)* is one of the most important cereal crops worldwide, serving as a source of animal feed or primary ingredient in the distillation and brewing industry (Baik and Ullrich 2008; Farag et al. 2022). During the last decades, the annual global production of barley has been hampered by various biotic stresses due to climate change, and among biotic stresses, scald is one of the major foliar diseases, reducing barley yield by up to 40% in susceptible varieties (McLean and Hollaway 2018). Due to ineffective management practices and climate instability, the incidence of scald disease caused by *Rhynchosporium secalis* has been increasing over the past years (Ababa et al. 2023). Scald is one of the major biotic constraints to barley production in high‐rainfall regions (Turkington et al. 2005; Paulitz and Steffenson 2010), which induces water‐soaked lesions on the leaf blades and sheaths and ultimately results in leaf necrosis, imposing a detrimental impact on photosynthesis, grain quality and yield (Zhan et al. 2008; Wondimu et al. 2022). The disease is currently controlled by applying fungicides; however, the extensive and repeated use of fungicides has resulted in the emergence of resistant pathogen strains, making the problem even more severe. For example, a recent global population‐genomic analysis of 125 isolates identified a large segmental duplication involving *CYP51A* that contributed to the emergence of azole resistance, demonstrating the capacity of this pathogen to adapt to fungicide use (Stalder et al. 2023). Therefore, there is an urgent need for more sustainable strategies to combat the emerging plant foliar disease (Avrova and Knogge 2012; Islam et al. 2024).

Host or varietal resistance represents one of the most effective and sustainable strategies for managing barley scald, particularly given the limitations associated with repeated fungicide applications. Considerable genetic variation for resistance to *R. secalis* has been identified in barley germplasm, with several major resistance loci (e.g., *Rrs1*, *Rrs2* and *Rrs13*) characterised and mapped in barley (Hanemann et al. 2009; Looseley et al. 2020; Eckstein et al. 2024). More recent genome‐wide association studies further identified numerous QTL and novel genomic regions associated with seedling and adult‐plant scald resistance (Hiddar et al. 2023; Noe et al. 2025). Despite this progress in identifying resistant germplasm and resistance‐associated loci, the physiological and molecular mechanisms underlying contrasting varietal responses to *R. secalis* remain less well understood. In particular, relatively little is known about how stomatal regulation, ion homeostasis, oxidative stress responses and defence‐related signalling networks are coordinated in resistant barley genotypes following pathogen infection. Molecular basis of pathogen resistance revealed critical cellular receptors in recognizing pathogen‐derived molecules (Kourelis and Van Der Hoorn 2018; Dodds et al. 2024). This includes pattern recognition receptors (PRRs) that can detect pathogenic molecules such as pathogen‐associated molecular patterns (PAMPs) and cellular damage signals during pathogen infections through wall‐associated kinases (WAKs) (Couto and Zipfel 2016). This recognition activates intracellular signalling cascades, involving ROS production, defence‐responsive gene expression and stomatal closure, which together contribute to early immune responses against pathogen invasion (Bakhoum et al. 2023; Ngou et al. 2022; Thabet et al. 2025). These immune responses are tightly regulated by important signalling proteins such as Enhanced Disease Susceptibility 1 (EDS1) and non‐race‐specific Disease Resistance 1 (NDR1). These proteins transduce defence signals from different types of resistance (*R*) genes inside the plant cell, helping to activate strong and effective defence reactions against the pathogen (McNeece et al. 2017).

Stomata play a major role in plant immunity, as they act as pathogens' points of access (Meddya et al. 2023). Stomatal morphology (e.g., stomatal density, size) and stomatal dynamics are closely associated with pathogen invasion, as pathogens often exploit stomatal openings to enter leaf tissues. Previous studies have shown that smaller stomata or more rapid stomatal closure can enhance plant resistance by restricting pathogen entry and enabling plants to rapidly perceive and respond to pathogen‐associated molecular patterns (PAMPs) (Melotto et al. 2006; Zeng and He 2010; Noman et al. 2023). Furthermore, K^+^ is one of the most important osmotic solutes in guard cells and plays a central role in regulating stomatal aperture by modulating guard cell turgor through K^+^ influx and efflux across the plasma membrane (Schroeder et al. 2001). In guard cells, Ca^2+^‐dependent signalling interacts with ROS pathways to regulate ion‐channel activity and stomatal closure during pathogen responses (Schroeder et al. 2001; Noman et al. 2021; Riaz et al. 2022). The increase in Ca^2+^ levels is recognized by calcium‐binding proteins such as calmodulin (CaM), calcium‐dependent protein kinases (CDPKs) and calcineurin B‐like proteins (CBLs), which further activate a range of transcription factors responsible for defence gene expression (Reddy et al. 2011; Sun, Yang, et al. 2022).

Although scald is known to reduce barley yield and grain quality (Kavak 2004), the physiological and molecular traits associated with *R. secalis* resistance remain insufficiently understood. In particular, it is unclear whether resistant barley germplasm maintains disease resistance through coordinated responses involving stomatal regulation, ion homeostasis, antioxidant capacity and defence‐related gene expression. We hypothesized that scald resistance in barley is associated with stable stomatal behaviour, K^+^ and Ca^2+^ homeostasis, enhanced ROS‐scavenging capacity and activation of defence‐related signalling genes following pathogen inoculation. To test this hypothesis, we compared a scald‐susceptible cultivar, Atlantis and a resistant cultivar, Yangsimai 3, under control and scald‐inoculated conditions. The objectives of this study were to: (i) compare growth and photosynthetic responses to *R. secalis* infection; (ii) determine whether stomatal traits and K^+^/Ca^2+^ status differ between susceptible and resistant germplasm after inoculation; (iii) assess oxidative stress and antioxidant enzyme responses and (iv) examine expression patterns of selected genes associated with antioxidant defence, ion transport, stomatal regulation, Ca^2+^ signalling and phytohormone‐mediated defence. This framework allowed us to identify physiological and molecular traits associated with scald resistance without implying direct causal relationships.

## 2 Materials and Methods

### 2.1 Plant Material

Two barley ( *Hordeum vulgare* ) cultivars, Atlantis (also known as RGT Atlantis, RAGT, AU) and Yangsimai 3 (a Chinese land race, highly resistant to scald), were used in this study. Atlantis is an Australian barley cultivar highly susceptible to scald. In contrast, Yangsimai 3 is a resistant cultivar that develops little or no lesions following pathogen inoculation and has been used as a resistance donor in barley breeding programs. Therefore, these two genotypes are not near‐isogenic lines (NILs) but represent contrasting germplasm suitable for investigating the physiological and molecular mechanisms underlying scald resistance. Mature barley seeds were surface‐sterilized in 70% ethanol for 5 min, followed by three rinses with distilled water. Sterilized seeds were then transferred to plastic pots (18 × 19 cm) filled with commercial potting mixture (Searles Garden product), with 4 seeds per pot. The experiment was conducted in a glasshouse at Mount Pleasant Laboratory (University of Tasmania, Launceston, TAS, 7250, Australia) from March to June 2025, with temperatures ranging from 18°C to 24°C and relative humidity of approximately 60%–80%. *Rhynchosporium secalis* inoculation was conducted when seedlings reached the three‐leaf stage (~20 days after germination), following the procedure described in Section 2.2. At least three independent biological replicates were used for each cultivar × treatment combination, with multiple plants or microscopic fields measured per replicate depending on the trait.

### 2.2 Scald Pathogen Propagation and Inoculation

Barley leaves infected with *R. secalis*, showing typical scald symptoms, were collected from naturally infected barley plants grown at the Mt. Pleasant laboratory field site, University of Tasmania, Launceston, Tasmania, Australia (approximately 41.4681° S, 147.1415° E), and used as the initial pathogen source for *R. secalis* propagation. Firstly, the infected leaf was surface‐sterilized by soaking in 70% ethanol for 1 min, followed by rinsing thrice with sterile distilled water. Afterwards, the leaf tissues were gently cleaned using Whatman filter paper to remove excess water. Leaves were then placed in a Petri dish with the infected side facing the potato dextrose agar (PDA) plates prepared by adding 20 g of PDA (granulated, HiMedia Laboratories Pvt. Ltd.) to 100 mL of distilled water and autoclaved (sterilisation holding time of 15 min under 121°C). The PDA plate with the pathogen was then incubated at 18°C for 1 week in the dark (Dhakal 2023). After darkness incubation, a fine glass needle was used to collect small tufts of mycelia that emerged around the lesion borders and transferred them to Lima bean agar (LBA) medium for sporulation under darkness at 18°C for 2 weeks. For the LBA medium, 25 g of lima beans were initially boiled in 1 L of distilled water for 20 min and then filtered through filter papers to remove residues. After filtration, 200 mL of the residue‐free lima bean extract was transferred to a flask, and 4 g of agar was added. The LBA medium was then autoclaved, with a sterilisation holding time of 15 min under 121°C, before being poured into Petri dishes to form solid LBA medium for sporulation.

**FIGURE 1:**
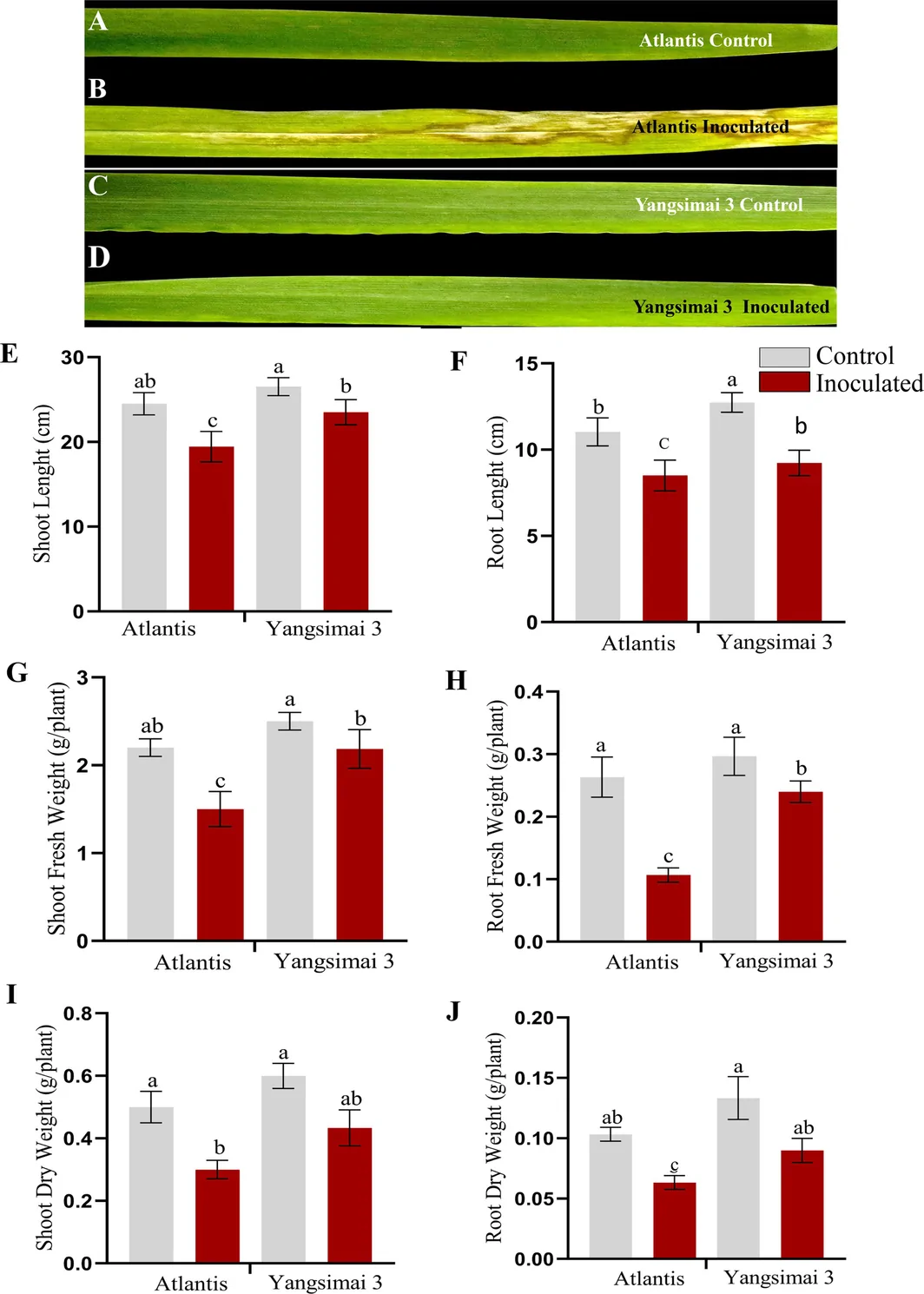
Comparison of scald infection among Atlantis and Yangsimai 3 and its effects on barley growth parameters. (A) Atlantis control; (B) Atlantis inoculated with the scald pathogen showing water‐soaked lesions on leaf blades; (C) Yangsimai 3 control; (D) Yangsimai 3 inoculated plants showing no visible disease symptoms. Growth parameters including (E) shoot length, (F) root length, (G) shoot fresh weight, (H) root fresh weight, (I) shoot dry weight and (J) root dry weight were measured. Data are presented as mean ± SD of three biological replicates (*n* = 3). Different lowercase letters indicate significant differences among cultivar × treatment combinations based on two‐way ANOVA followed by Tukey's HSD test (*p* ≤ 0.05).

After 2 weeks of incubation, 10 mL of sterile distilled water was added to each sporulating LBA plate, and the conidia were gently dislodged using sterile cotton swabs. The resulting conidial suspension was filtered through two layers of sterile cheesecloth to remove mycelial fragments and agar debris. The conidial concentration was not quantitatively determined during the experiment. Barley seedlings at the fully developed three‐leaf stage, approximately 20 days after sowing, were inoculated by spraying the conidial suspension onto the foliage using an atomizer until the leaf surfaces were uniformly wetted. Uninoculated control plants were sprayed with sterile water using the same atomizer and maintained under the same post‐inoculation conditions. Immediately after inoculation, plants were completely covered with cling wrap and maintained at approximately 100% relative humidity in darkness for 48 h to facilitate infection. After *R. secalis* inoculation, infected plants were transferred to a glasshouse with a temperature of 16°C night/20°C day under natural light and 80%–90% relative humidity. Disease symptoms appeared around the four‐leaf stage (approximately 7 days after inoculation), when most physiological traits were investigated; barley plants were visually assessed after scald based on the presence and severity of water‐soaked and necrotic lesions on leaf blades and sheaths. The contrasting disease responses of Atlantis and Yangsimai 3 were recorded photographically and used to confirm the susceptible and resistant phenotypes (Figure 1).

### 2.3 Measurement of Growth Parameters

Barley plants were uprooted with intact shoots and roots at the four‐leaf stage and washed with water to remove adhered potting mix particles. The plant height was recorded using a measuring scale. The fresh and dry weights (oven‐dried at 80°C for 3 days; until constant weight) of plants were measured using an electronic weighing balance. Three plants per replication and three replicates per treatment were measured.

### 2.4 Chlorophyll, Carotenoid Contents, Stomatal Conductance and Stomatal Morphological Traits Measurements

SPAD (Soil Plant Analysis Development) values, a proxy for non‐destructive measurement of chlorophyll content, were recorded from a fully expanded second‐top leaf using a portable SPAD‐502+ device (Tokyo). Stomatal conductance was measured on the same expanded leaf using Li‐6400XT under 1000 μmol m^−2^ s^−1^ PAR, 25°C, 50% relative humidity, 500 μmol m^−2^ s^−1^ airflow and~ 1.5 VPDL. Stomatal traits were measured following previously described methods with minor modifications (Wu and Zhao 2017). Briefly, fully expanded leaves from barley seedlings at around four‐leaf stage were sampled. Epidermal imprints were obtained from the abaxial leaf surface of a second‐top fully expanded leaf using clear nail polish. Imprints were carefully peeled off and mounted on microscope slides. Images were captured using a light microscope under 400× magnification. For each leaf epidermal imprint, at least five non‐overlapping microscopic fields were randomly selected and photographed. These images were used to quantify stomatal density, stomatal index and stomatal pore size using ImageJ software. Three independent plants were used as biological replicates for each genotype × treatment combination. For stomatal size traits, at least 40 clearly visible stomata per plant were analysed from non‐overlapping microscopic fields using ImageJ software (Collins 2007; Guo et al. 2024). Stomatal index (SI) was calculated using the formula: SI = [*S*/*S*(*S* + *E*)], where S represents the number of stomata and E represents the number of epidermal cells within the same microscopic field (Royer 2001). Furthermore, barley plants were harvested with intact shoots and roots, washed with tap water carefully for the measurement of shoot and root length, and fresh and dry biomass. For carotenoid measurement, plants were harvested at the four‐leaf stage, when disease symptoms were clearly observable following pathogen inoculation. 100 mg leaf sample with three biological replicates per treatment was homogenized in 80% methanol and centrifuged for 15 min at 15000 × g. The supernatant was collected in a separate tube, and absorbance was measured at 480 nm using a spectrophotometer and carotenoid content was calculated according to Garzón et al. (2012) based on absorbance (*A*_480_), extraction volume (*V*), dilution factor (DF) and fresh sample weight (*W*), and expressed as μg g^−1^ FW following formula: $\text{Carotenoid content}=\frac{A_{480}\times V\times \mathrm{DF}}{W}.$

### 2.5 Estimation of H_2_O_2_ , MDA Production and Antioxidant Enzymes Content

MDA and H_2_O_2_ contents were measured using fully expanded leaves collected from control and scald‐inoculated barley seedlings at about four‐leaf stage. For inoculated plants, leaves showing visible scald symptoms were sampled from susceptible Atlantis plants, while corresponding leaves at the same developmental stage were collected from Yangsimai 3 and control plants. For each measurement, three biological replications were conducted. Briefly, a 0.2 g flag leaf sample was homogenized in 2 mL of 65 mM potassium phosphate buffer (pH 7.8) and centrifuged at 2600 × g for 10 min at 4°C. For H_2_O_2_ measurement, 1 mL of the supernatant was mixed with 0.1 mL of 10 mM hydroxylamine hydrochloride and 0.9 mL of 65 mM potassium phosphate buffer (pH 7.8) and incubated at 25°C for 20 min. After the incubation, 1 mL of 17 mM 4‐aminobenzenesulphonic acid (C_6_H_7_NO_3_S) and 1 mL of 7 mM α‐naphthylamine (C_10_H_7_NH_2_) were added. The solution was gently shaken and incubated for an additional 20 min at 25°C. 3 mL of trichloromethane (CHCl_3_) was added to the samples, and the absorbance of the samples was recorded at 530 nm (Guo et al. 2026). The Malondialdehyde (MDA) content was determined according to Heath and Packer (1968). 0.1 g of flag leaf plant tissue was pre‐chilled with 1 mL potassium phosphate buffer (65 mM; pH 7.8) and centrifuged at 15000 × g at 4°C for 15 min. The supernatant was mixed with 1 mL of 0.5% thiobarbituric acid (TBA) and incubated at 95°C for 25 min. Afterwards, the samples were placed on ice to stop the reaction, and MDA contents were measured using a UV–visible spectrophotometer at 532 nm (Heath and Packer 1968).

For measuring the activity of ROS scavenging‐related enzymes, fully expanded fresh seedling leaf samples (0.5 g) were homogenized in 2 mL of ice‐cooled 50 mM sodium phosphate buffer (pH 7.8), centrifuged at 18000 × g for 20 min at 4°C, for the determination of antioxidant enzymes peroxidase (POD; EC 1.11.1.7), superoxide (SOD; EC 1.15.1.1) and catalase (CAT; EC 1.11.1.6). The enzyme activities were spectrophotometrically measured at 470 nm for POD (Chen et al. 2010), 520 nm for CAT (Kar and Mishra 1976) and 560 nm for SOD (Zhang et al. 2008), and expressed as U mg^−1^ protein for comparison.

### 2.6 Measurement of Ca^2+^ and K^+^ Content in Barley Leaves

Fresh leaf samples were collected from both control and *R. secalis*‐inoculated plants at around the four‐leaf growth stage, with three biological replicates per treatment. The samples were oven‐dried at 80°C until constant weight to remove moisture content before measurement of potassium (K^+^) and calcium (Ca^2+^) concentrations. Briefly, 100 mg of dried leaf samples were weighed, cut into smaller pieces and put into 15 mL digestion tube. 10 mL of acetic acid (100 mM) was added to each tube and heated at 90°C for 5 h. All samples were inverted repeatedly at 30‐min intervals to ensure uniform digestion of the leaf samples. After that, samples were removed and allowed to cool at room temperature. 1 mL of the digestion sample was taken to measure the K^+^ and Ca^2+^ concentrations using a portable meter (HORIBA Advanced Techno Co. Ltd).

### 2.7 qRT‐PCR Analysis

To capture the most relevant pathogen‐responsive gene expression changes, fresh leaf samples were collected 2 days post‐*R. secalis* inoculation for qPCR analysis. Approximately 100 mg of fresh tissue with three biological replicates was weighed and finely ground into a powder using liquid nitrogen with a mortar and pestle. RNA was then extracted using the RNeasy Plant mini‐Kit (Qiagen), following the manufacturer's instructions. The concentration and quality of isolated RNA were quantified by Thermo Nanodrop 2000 (Thermo Fisher Scientific). All‐in‐One First‐Strand synthesis kit (Thermo Fisher Scientific) was used to synthesize cDNA by taking 1 μL of RNA sample as a template. The online tool “Oligo Calculator” (http://mcb.berkeley.edu/labs/krantz/tools/oligocalc.html) was used to design gene‐specific primers. The qRT‐PCR analysis was carried out by a CFX96 real‐time PCR system (Bio‐Rad) with iTaq qPCR SYBR Universal Green Master Mix (Bio‐Rad). The thermal program for the quantitative PCR amplification was 95°C for 30 s, 95°C for 15 s and 60°C for 30 s, repeated for 40 cycles; the melting curve was from 65°C to 95°C. 2^−∆∆CT^ (*C*_T_, threshold cycle) method was employed to measure the relative expression of each gene (Livak and Schmittgen 2001) using the *ACTIN* gene (Table S1) as a housekeeping gene to normalize the data (Ferdous et al. 2015).

### 2.8 Statistical Analysis

Data were analysed in R (R 4.4.3) using two‐way ANOVA. Linear models were fitted using the lm() function, with cultivar (Atlantis and Yangsimai 3) and treatment (uninoculated control and *R. secalis*‐inoculated) as fixed factors, and the cultivar × treatment interaction was also tested. Prior to ANOVA, data were checked for normality and homogeneity of variance using the Shapiro–Wilk and Levene's tests, respectively. When significant main effects or interactions were detected, means were compared using Tukey's honestly significant difference (HSD) test at *p* ≤ 0.05. When significant main effects or interactions were detected, means were compared using Tukey's honestly significant difference (HSD) test at *p* ≤ 0.05. Data are presented as mean ± standard deviation (SD) of three independent biological replicates unless otherwise stated. Principal component analysis (PCA) was performed in R using the prcomp() function. The variables included in the PCA were shoot and root length, shoot and root fresh weight, shoot and root dry weight, SPAD value, stomatal conductance, stomatal density, stomatal index, stomatal pore size, K^+^ and Ca^2+^ contents, H_2_O_2_ and MDA contents, and SOD, POD and CAT activities. Prior to PCA, all variables were Z‐score standardized using the scale() function in R. The PCA biplot was visualized using the factoextra and ggplot2 packages, and the heatmap was generated using the pheatmap package.

## 3 Results

### 3.1 Scald Infection Causes Genotype‐Dependent Reductions in Growth and Photosynthetic Performance

Two‐way ANOVA revealed significant cultivar × treatment interactions for shoot length (*p* = 0.002), root length (*p* = 0.031), shoot fresh weight (*p* = 0.040) and root fresh weight (*p* = 0.004), indicating that the effects of *R. secalis* inoculation on these growth traits differed between Atlantis and Yangsimai 3 (Table S2). In contrast, the cultivar × treatment interactions were significant for growth and photosynthetic traits, except shoot dry weight, SPAD value and chlorophyll fluorescence (*p* > 0.05). At 14 days post‐inoculation, Atlantis showed severe disease symptoms, including water‐soaked and necrotic lesions on the leaf blades and sheaths, whereas Yangsimai 3 showed only slight or no visible lesions (Figure 1A–D). Relative to the uninoculated Atlantis control, *R. secalis* inoculation reduced shoot and root length by 20.6% and 22.9%, respectively, and shoot and root fresh weight by 31.8% and 19.4%, respectively. These reductions were less pronounced in Yangsimai 3 (Figure 1E–H), indicating that the effect of scald inoculation differed between Atlantis and Yangsimai 3. After 14 days of inoculation, *R. secalis* inoculation in Atlantis caused severe symptoms of infection, water‐soaked lesions on the leaf blades and sheaths, along with restrained growth of shoot and root length (Figure 1A,B), while causing slight or no scald lesions in Yangsimai 3 compared to nonincubated control plants (Figure 1A–D).

**FIGURE 2:**
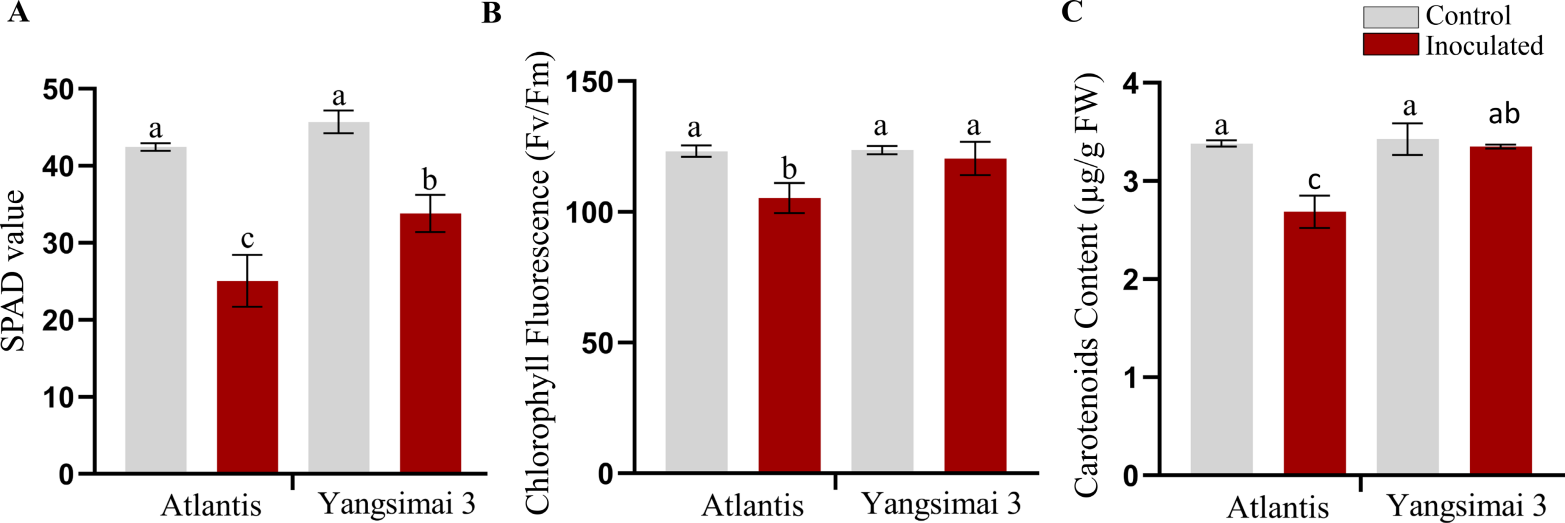
Effects of scald infection on photosynthetic parameters among two genotypes. (A) SPAD value, (B) chlorophyll fluorescence and (C) carotenoid contents in Atlantis and Yangsimai 3 under control and scald‐inoculated conditions. Data are presented as mean ± SD of three biological replicates (*n* = 3). Different lowercase letters indicate significant differences among cultivar × treatment combinations based on two‐way ANOVA followed by Tukey's HSD test (*p* ≤ 0.05).

Additionally, scald infection caused differential effects on SPAD, chlorophyll fluorescence and carotenoid contents between the two cultivars. This genotype‐dependent response was most evident in Atlantis, where SPAD and chlorophyll fluorescence decreased by 30.9% and 10.8%, respectively, after *R. secalis* inoculation (Figure 2A,B). In contrast, *R. secalis* infection only had a very minor effect on SPAD and chlorophyll fluorescence values in Yangsimai 3 (Figure 2B). However, the cultivar × treatment interactions were not significant for either SPAD (*p* = 0.0652) or chlorophyll fluorescence (*p* = 0.6128; Table S2). Carotenoid content was also reduced in Atlantis by *R. secalis* inoculation (by 18.6% as compared to the non‐inoculated control plants; *p* < 0.01), while Yangsimai 3 maintained a relatively high carotenoid level post *R. secalis* inoculation (Figure 2C). Collectively, Yangsimai 3 possessed much higher tolerance to scald infection than the Atlantis plants.

### 3.2 Stomatal Responses to Scald Infection Differ Between Atlantis and Yangsimai 3

**FIGURE 3:**
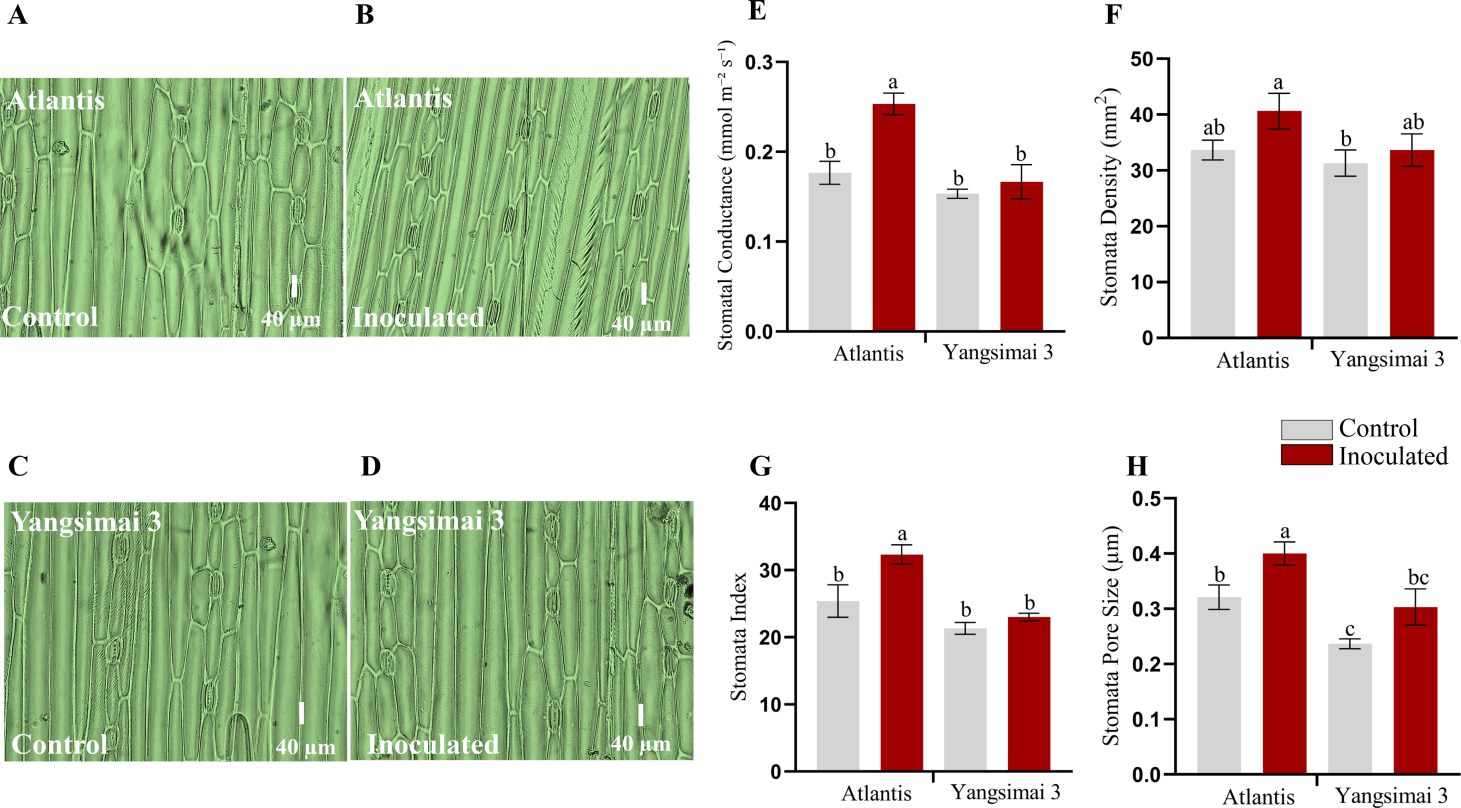
Comparison of stomatal morphological traits among barley genotypes. (A–D) Representative microscopic images of stomata in Atlantis (susceptible cultivar) and Yangsimai 3 (resistant cultivar) under control and scald‐inoculated conditions. (A) Atlantis control, (B) Atlantis inoculated, (C) Yangsimai 3 control and (D) Yangsimai 3 inoculated. Scale bars = 40 μm. (E) Stomatal conductance, (F) stomatal density, (G) stomatal index and (H) stomatal pore size measured in Atlantis and Yangsimai 3 plants under control and scald‐inoculated conditions. Data are presented as mean ± SD of three independent biological replicates (*n* = 3). Different lowercase letters indicate significant differences among cultivar × treatment combinations based on two‐way ANOVA followed by Tukey's HSD test (*p* ≤ 0.05).

Stomata play a critical role in defence against fungal infections in plants. Upon pathogen attack, plants actively close their stomata to restrict pathogen entry and colonisation into host tissues (Melotto et al. 2006). *R. secalis* inoculation increased stomatal conductance in Atlantis, whereas Yangsimai 3 maintained relatively stable conductance. A significant cultivar × treatment interaction was detected for stomatal conductance (*p* = 0.0009) and stomatal pore size (*p* = 0.0083), whereas the interactions were not significant for stomatal index (*p* = 0.1171) or stomatal density (*p* = 0.3963; Table S2; Figure 3E–H).

### 3.3 K^+^ and Ca^2+^ Contents Are More Stable in Yangsimai 3 Under *R. secalis* Infection

**FIGURE 4:**
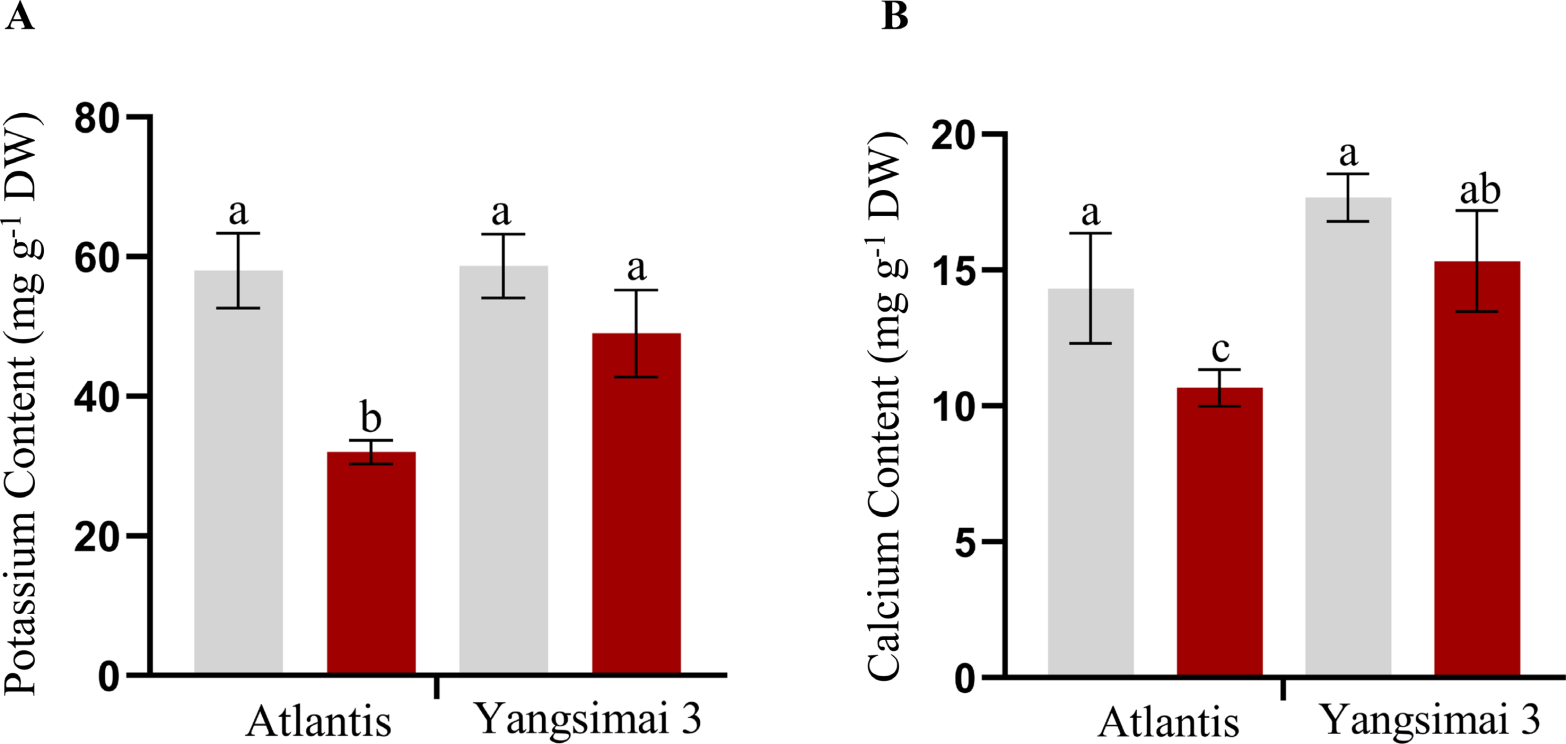
Effects of scald infection on potassium and calcium contents in barley leaves. (A) Potassium content and (B) calcium content in Atlantis and Yangsimai 3 under control and scald‐inoculated conditions. Data are presented as mean ± SD of three independent biological replicates (*n* = 3). Different lowercase letters indicate significant differences among cultivar × treatment combinations based on two‐way ANOVA followed by Tukey's HSD test (*p* ≤ 0.05).

Under non‐inoculated control conditions, no statistical difference was observed regarding K^+^ and Ca^2+^ among the two genotypes, even though Yangsimai 3 possessed slightly higher Ca^2+^ content than Atlantis (Figure 4). *R. secalis* inoculation reduced K^+^ and Ca^2+^ contents in Atlantis by 34.8% (*p* < 0.01) and 25.5% (*p* < 0.05), respectively, whereas Yangsimai 3 maintained relatively stable ion contents (Figure 4A,B).

### 3.4 Yangsimai 3 Shows Lower Oxidative Damage and Stronger Antioxidant Enzyme Responses Under Scald Infection

**FIGURE 5:**
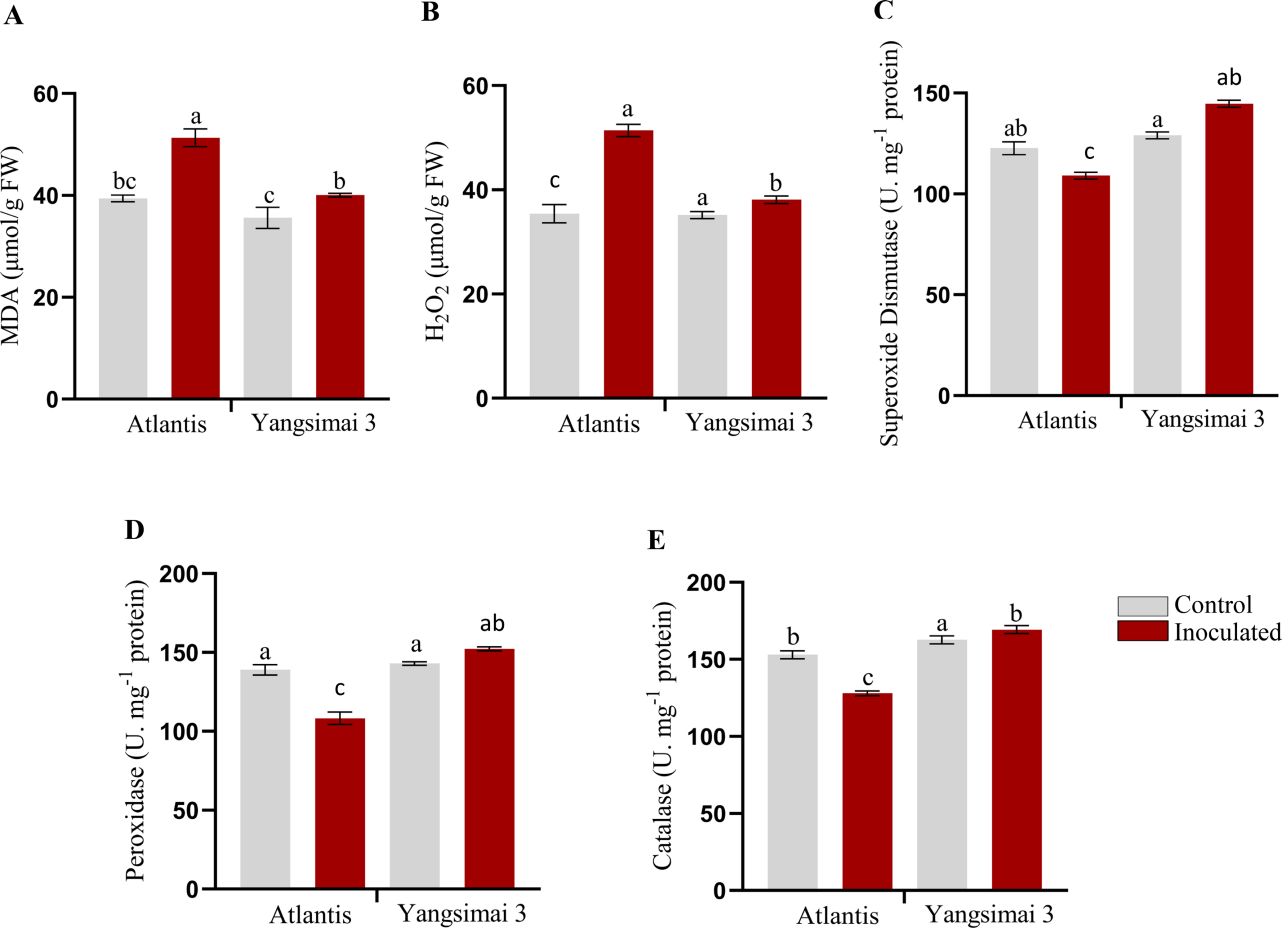
Effects of scald infection on oxidative stress markers and antioxidant enzyme activities in barley. (A) Malondialdehyde (MDA) content, (B) hydrogen peroxide (H_2_O_2_) content, (C) superoxide dismutase (SOD) activity, (D) peroxidase (POD) activity and (E) catalase (CAT) activity in Atlantis and Yangsimai 3 under control and scald‐inoculated conditions. Data are presented as mean ± SD of three independent biological replicates (*n* = 3). Different lowercase letters indicate significant differences among cultivar × treatment combinations based on two‐way ANOVA followed by Tukey's HSD test (*p* ≤ 0.05).

The H_2_O_2_ and MDA contents are common indicators of oxidative damage in plants under biotic stress. Scald infection induces oxidative stress in barley, resulting in excessive accumulation of ROS (Camejo et al. 2016). Scald inoculation increased H_2_O_2_ and MDA in Atlantis by 30.18% and 35.1%, respectively, but these oxidative stress markers remained lower in Yangsimai 3 (Figure 5A,B). Although H_2_O_2_ and MDA increased more strongly in Atlantis following *R. secalis* inoculation, the cultivar × treatment interactions were not significant for either MDA or H_2_O_2_ (*p* = 0.0879 for both; Table S2). In parallel, Yangsimai 3 showed higher anti‐oxidant enzyme activities (U mg^−1^ protein) than Atlantis after *R. secalis* inoculation, showing enhanced SOD, POD and CAT activity by 12.14%, 8.52% and 8.2%, respectively, compared to Atlantis plants, suggesting an active defence mechanism against oxidative stress (Figure 5C–E). Significant cultivar × treatment interactions were detected for SOD (*p* = 0.0002), POD (*p* = 0.0001) and CAT (*p* = 0.0002), supporting contrasting antioxidant responses between Atlantis and Yangsimai 3 following *R. secalis* inoculation (Table S2).

### 3.5 Defence‐Related Gene Expression Shows Genotype‐Dependent Responses to Scald Infection

**FIGURE 6:**
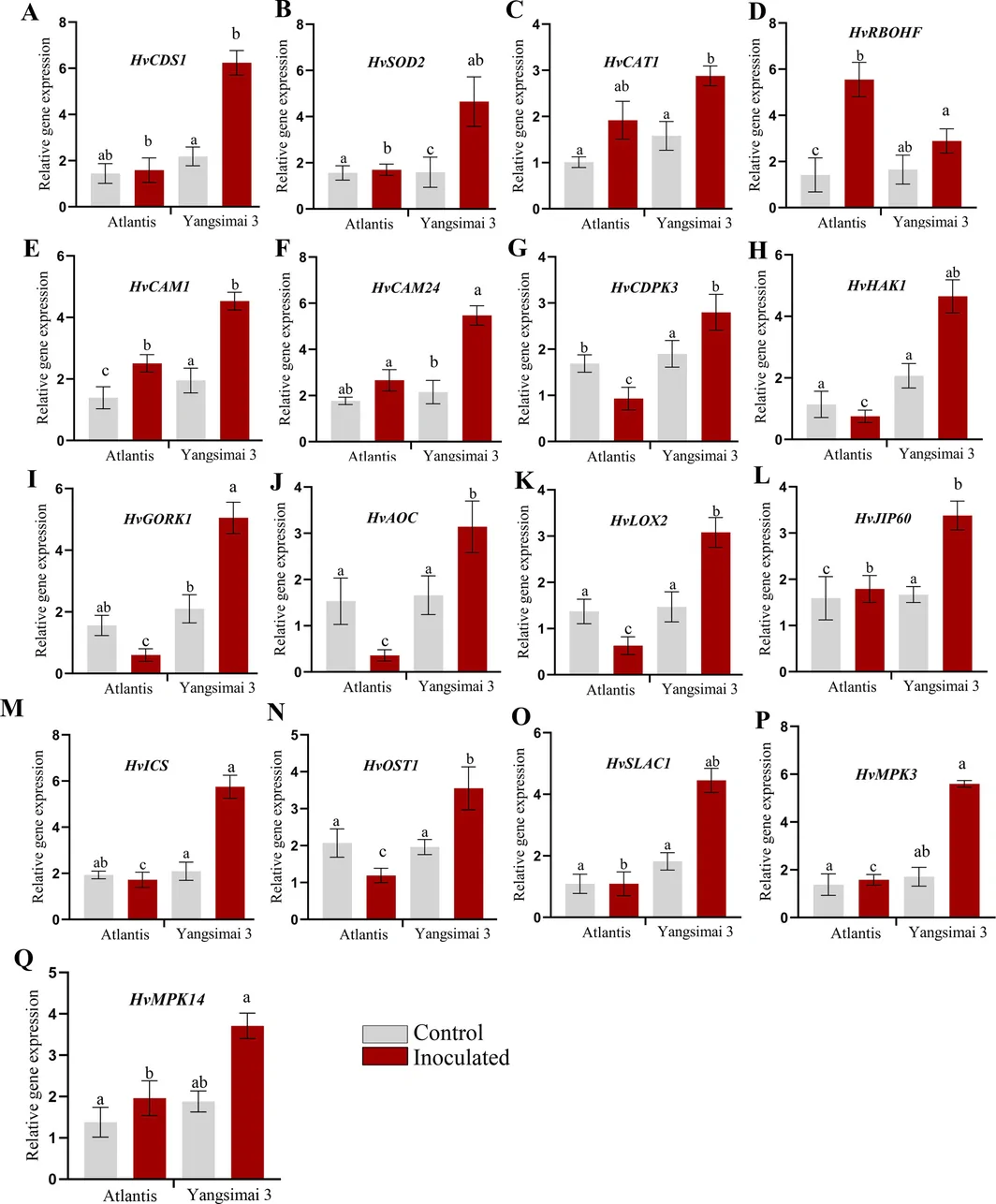
Expression profiles of defence‐related genes in barley under scald infection. Relative expression of genes involved in antioxidant defence, Ca^2+^ signalling, ion transport, phytohormone signalling and stomatal immunity in Atlantis and Yangsimai 3 under control and scald‐inoculated conditions. (A–C) Antioxidant‐related genes: *HvCDS1*, *HvSOD2*, and *HvCAT1*. (D) ROS production gene: *Hvrbohf*. (E–G) Ca^2+^ signalling genes: *HvCAM1*, *HvCAM24*, and *HvCDPK3*. (H–I) Ion transport genes: *HvHAK1* and *HvGORK1*. (J–L) JA biosynthesis genes: *Hvaoc*, *HvLOX2* and *HvJIP60*. (M) SA biosynthesis gene: Hvics. (N–O) Stomatal regulation genes: HvOST1 and HvSLAC1. (P–Q) MAPK signalling genes: *HvMPK3* and *HvMPK14*. Gene expression levels were determined by qRT‐PCR and normalized to the reference gene Actin. Data are presented as mean ± SD of three independent biological replicates (*n* = 3). Different lowercase letters indicate significant differences among cultivar × treatment combinations based on two‐way ANOVA followed by Tukey's HSD test (*p* ≤ 0.05).

To investigate transcriptional responses associated with scald resistance, representative genes related to antioxidant defence, ROS production, Ca^2+^ signalling, ion transport, phytohormone signalling, stomatal regulation and MAPK‐mediated immune signalling were investigated by qRT‐PCR using leaf tissues collected from control and *R. secalis*‐infected plants (Figure 6; Table S1). Overall, *R. secalis* inoculation induced genotype‐dependent expression patterns, with Yangsimai 3 showing stronger activation of multiple defence‐related pathways than Atlantis. Genes associated with antioxidant defence showed stronger induction in Yangsimai 3 following *R. secalis* inoculation. The expression levels of *HvSOD2*, *HvCDS1* and *HvCAT1* were markedly upregulated in Yangsimai 3 plants by 2‐, 2.5‐ and 1.5‐fold compared with their corresponding controls (Figure 6A–C). In contrast, the ROS‐production‐associated gene *HvRBOHF* showed stronger induction in Atlantis than in Yangsimai 3 after *R. secalis* inoculation, suggesting greater activation of ROS‐generating processes in the susceptible cultivar (Figure 6D). These transcriptional patterns are consistent with the lower H_2_O_2_ and MDA levels and higher antioxidant enzyme activities observed in Yangsimai 3 (Figure 5A,B). Genes involved in Ca^2+^ signalling and ion transport also differed between the two cultivars under *R. secalis* infection. In Yangsimai 3, *HvCAM1*, *HvCDPK3* and *HvCAM24* were upregulated, together with increased expression of K^+^ signalling genes *HvHAK1* and *HvGORK1* (Figure 6E–I). By Contrast, these ion transport‐related responses were weaker or downregulated in *Atlantis* post *R. secalis* inoculation (Figure 6E–I). These results suggest that Yangsimai 3 maintains a stronger transcriptional response associated with Ca^2+^ signalling and ion homeostasis under scald infection.

Phytohormone‐related genes also showed cultivar‐specific responses. The JA‐associated genes *HvAOC*, *HvLOX2* and *HvJIP60*, together with the SA biosynthesis‐related gene *HvICS*, were more strongly induced in Yangsimai 3 than in Atlantis after *R. secalis* inoculation (Figure 6J–M). In Atlantis, *HvAOC* and *HvLOX2* were downregulated after *R. secalis* inoculation, while only a moderate increase in *HvJIP60* expression was observed. These results indicate that Yangsimai 3 may activate stronger JA‐ and SA‐related defence signalling responses under *R. secalis* infection. Similarly, genes associated with stomatal regulation and MAPK‐mediated immune signalling showed stronger induction in Yangsimai 3. The expression of *HvOST1, HvSLAC1*, *HvMPK3 and HvMPK14* was substantially upregulated in Yangsimai 3, whereas their induction was weaker in Atlantis in response to *R. secalis* inoculation (Figure 6N–Q). Taken together, these qRT‐PCR results indicate that the resistant cultivar Yangsimai 3 exhibits stronger transcriptional activation of genes associated with antioxidant defence, Ca^2+^ signalling, ion transport, phytohormone signalling, stomatal regulation and immune signalling. These expression patterns are associated with the contrasting physiological responses observed between Yangsimai 3 and Atlantis under *R. secalis* infection.

### 3.6 Principal Component Analysis (PCA) Summarizes Trait Associations Among Cultivar × Treatment Combinations

**FIGURE 7:**
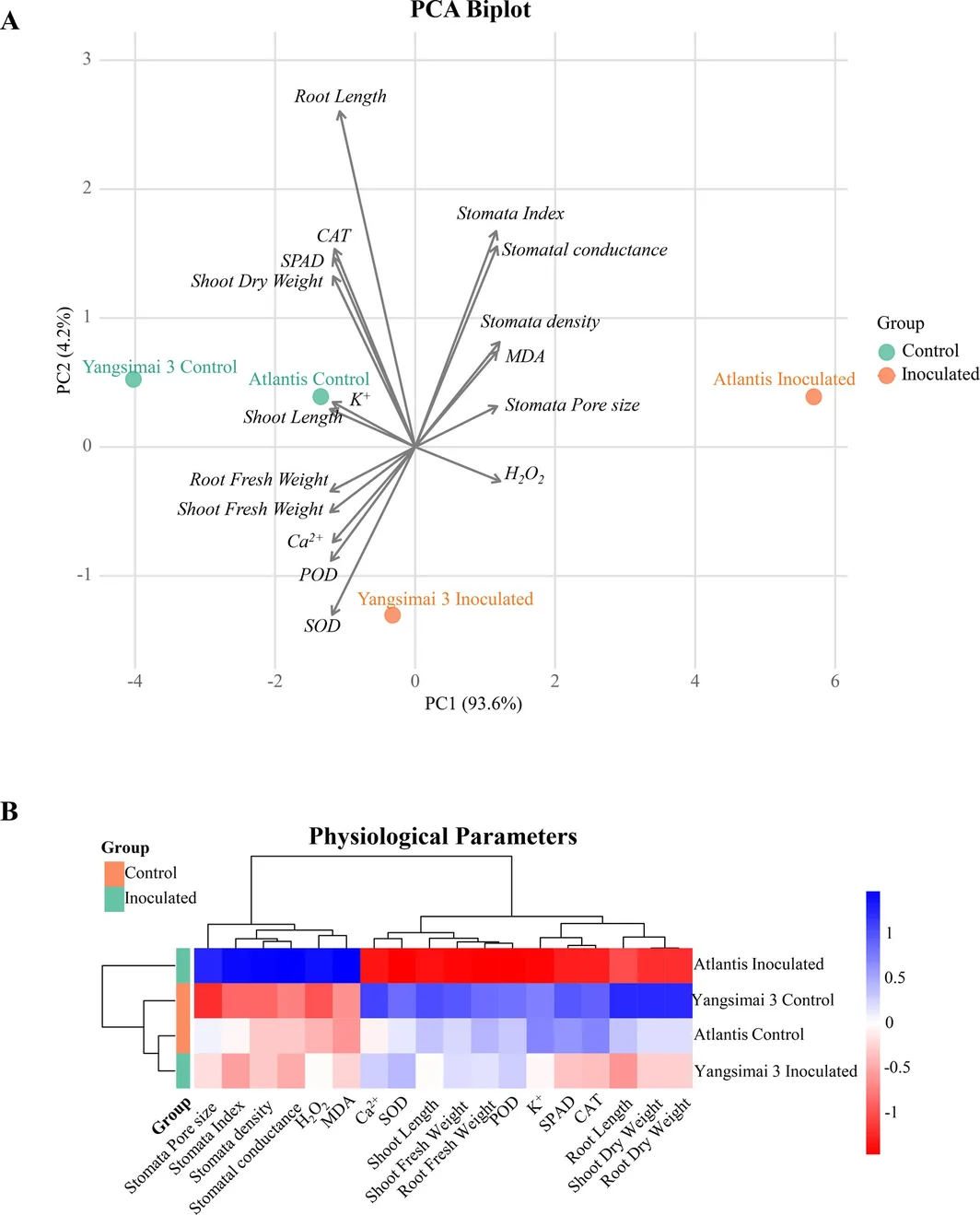
Multivariate analysis of physiological and biochemical responses to scald infection in barley. (A) Principal component analysis (PCA) biplot showing the distribution of physiological and biochemical variables and treatment groups in Atlantis and Yangsimai 3 under control and scald‐inoculated conditions. (B) Hierarchical clustering heatmap illustrating the relative responses of physiological and biochemical parameters across different treatments. Data were normalized using Z‐score transformation. Red and blue colours represent higher and lower values, respectively. All variables were Z‐score standardized before PCA and heatmap analysis. PCA was used as an exploratory approach to summarize overall trait variation among cultivar × treatment combinations.

PCA was performed using Z‐score standardized physiological and biochemical variables to explore overall patterns of trait variation among the four cultivar × treatment combinations (Figure 7A). PC1 explained 93.6% of the total variance, while PC2 explained 4.2%. The high contribution of variance explained by PC1 mainly reflected the strong separation of *R. secalis*‐inoculated Atlantis from the other groups, consistent with coordinated changes in growth, photosynthetic parameters, ion contents, stomatal traits, antioxidant enzyme activities and oxidative stress markers. Traits associated with better growth performance, photosynthetic status, ion maintenance and antioxidant capacity, including biomass‐related traits, SPAD value, K^+^ content, SOD, POD and CAT activities, were positioned on the side of the PCA plot closer to Yangsimai 3 under scald inoculation (Figure 7A). In contrast, oxidative stress markers, including H_2_O_2_ and MDA, were positioned in the opposite direction and were more closely associated with *R. secalis*‐inoculated Atlantis (Figure 7A). These patterns indicate that Yangsimai 3 maintained a more favourable physiological and biochemical profile under scald infection, whereas Atlantis showed stronger oxidative stress and growth inhibition.

The heatmap analysis further supported this separation pattern (Figure 7B). Scald‐inoculated Yangsimai 3 clustered with relatively higher values for growth‐related traits, ion contents and antioxidant enzyme activities, while *R. secalis*‐inoculated Atlantis was characterized by higher oxidative stress markers and less favourable growth‐related responses. Therefore, PCA and heatmap analyses were interpreted as exploratory tools summarizing trait associations among cultivar × treatment combinations, rather than as evidence of causal relationships.

## 4 Discussion

### 4.1 Scald‐Induced Disruption of Plant Growth Is Linked With Reduced Photosynthetic Activity in Atlantis

Previous study has shown that scald infections impair the operation of photosystem II (PSII), reduce chlorophyll content and decrease chlorophyll fluorescence efficiency (Fv/fm values), ultimately leading to reduced photosynthetic performance and plant growth (Hwang et al. 2006; Hoffman et al. 2014; Yahya et al. 2020). In Atlantis, stronger growth inhibition was accompanied by more severe necrotic lesion development and greater reductions in photosynthetic parameters (Figure 2A,B). Previous studies have also suggested that foliar necrotic lesions may impair photosynthetic performance by reducing functional leaf area and affecting chloroplast‐associated processes (Cheng et al. 2016; Brugger et al. 2018), but chloroplast structural changes were not directly examined. In our study, Yangsimai 3 maintained relatively stable photosynthetic performance and growth under *R. secalis* infection, suggesting that the maintenance of photosynthetic capacity may contribute to its enhanced resistance (Figure 2A–C).

Carotenoids are crucial photosynthetic pigments that protect plants against photo‐oxidative damage and play key roles in the light‐harvesting complex. Moreover, carotenoids function as non‐enzymatic antioxidants capable of scavenging reactive oxygen species (ROS), including hydroxyl radicals (Swapnil et al. 2021; El‐Awadi et al. 2025). Therefore, the smaller reduction in photosynthetic capacity in Yangsimai 3 may be attributed to reduced oxidative damage and sustained electron transport efficiency within PSII, which helps maintain photosynthetic activity under pathogen attack (Cheaib and Killiny 2024). The relatively stable photosynthetic performance of Yangsimai 3 is consistent with its lower disease symptoms, but this relationship should be interpreted as an association rather than direct evidence that photosynthetic stability determines resistance. Further studies using additional genotypes or controlled manipulation of photosynthetic capacity would be needed to test this relationship more directly.

### 4.2 Stomatal Closure in Yangsimai 3 Likely Contributes to Enhanced Scald Resistance

To further understand the physiological basis of scald resistance, we examined stomatal traits that regulate pathogen entry into leaf tissues. Stomatal closure represents an important pre‐invasive defence mechanism that limits pathogen entry into plant tissues (Zeng et al. 2010; Wu and Liu 2022; Noman et al. 2023). For example, Noman et al. (2023) showed that watermelon plants treated with *Acidovorax citrulli* (*Ac*) stimulate stomatal closure to restrict entry of *Ac* into the leaves. Recent studies have also shown that stomatal responses during pathogen infection are regulated by multiple signalling molecules, including ROS, SA and ABA (Bharath et al. 2021).

Among these pathways, ABA signalling plays a central role in regulating stomatal movement. Upon pathogen perception, ABA binds to RCAR/PYL receptors, which activates the protein kinase OPEN STOMATA 1 (OST1). OST1, a member of the SnRK2 family, stimulates stomata closure by phosphorylating ion channels such as H^+^‐ATPases, K^+^ transporters and slow ion channel 1 (SLAC1), thereby inducing stomatal closure (Umezawa et al. 2009; Hsu et al. 2021). Consistent with this mechanism, *Arabidopsis thaliana* *ost1* mutants exhibit impaired stomatal closure in response to pathogen‐associated molecular patterns such as *flg22* (Melotto et al. 2006). In addition, OST1 can promote ROS production through activation of NADPH oxidases, further reinforcing stomatal closure and plant defence responses. Other signalling components, including SA and Ca^2+^, also interact with ABA signalling to regulate stomatal dynamics during pathogen attack (Sun et al. 2020).

Similar observations have been reported in *Arabidopsis* plants inoculated with the multi‐host phytopathogen *Pseudomonas syringae* pv. tomato DC3000, where impaired stomatal closure facilitated pathogen entry (Gudesblat et al. 2009). In contrast, Yangsimai 3 exhibited reduced stomatal index, stomatal density and stomatal conductance following scald infection, suggesting a reduced probability of pathogen entry into leaf tissues (Figure 3E–H). Furthermore, Yangsimai 3 showed enhanced expression of *HvSLAC1* and *HvOST1* following *R. secalis* inoculation (Figure 6N,O), strongly suggesting active regulation of stomatal closure during infection. Similarly, *Brassica napus* infected with *Xanthomonas campestris* exhibits stomatal closure mediated by ROS and Ca^2+^ signalling pathways (Al‐Mamun et al. 2023). Likewise, activation of stomatal immunity has been associated with reduced disease severity in wheat infected with *Septoria tritici* (Battache et al. 2024). The lower stomatal conductance and smaller stomatal pore size observed in Yangsimai 3 after *R. secalis* inoculation are consistent with a potential role of stomatal regulation in limiting pathogen entry. However, because pathogen penetration through stomata was not directly quantified in this study, these stomatal traits should be interpreted as resistance‐associated features rather than direct evidence of reduced pathogen entry. Future microscopic tracking of pathogen penetration or manipulation of stomatal aperture would be required to confirm this proposed role. Additionally, although stomatal closure is commonly considered an early defence response, susceptible genotypes may fail to maintain effective stomatal regulation after pathogen challenge. The increased stomatal conductance observed in Atlantis may therefore reflect impaired guard‐cell regulation under scald infection rather than an adaptive defence response.

### 4.3 Enhanced ROS‐Scavenging Capacity Improves Scald Resistance in Yangsimai 3

In addition to restricting pathogen entry through stomatal regulation, plants must also mitigate oxidative stress generated during pathogen infection. Pathogen attack often triggers a rapid accumulation of ROS, which functions as an early defence signal but may also cause severe cellular damage when excessively accumulated. Plants continuously generate ROS as part of normal cellular metabolism to regulate various physiological processes and maintain homeostasis (Torres et al. 2006; Hassan et al. 2025). However, the role of ROS in plant pathogen interactions is complex and depends on the balance between ROS production and scavenging (Lehmann et al. 2015; Mittler et al. 2022). An initial ROS burst contributes to the activation of defence responses and restriction of pathogen growth (Moatamedi et al. 2023), whereas excessive ROS accumulation can lead to oxidative stress, resulting in cellular damage, impaired growth and disruption of defence signalling (Shetty et al. 2008; Khattab et al. 2024). To maintain ROS homeostasis, plants activate antioxidant defence systems involving enzymes such as SOD, POD and CAT, which detoxify ROS and restore redox balance after defence signalling is initiated (Qalavand et al. 2022; Dawood et al. 2024). Stronger activities of antioxidant enzymes, including SOD, POD and CAT were observed in Yangsimai 3 (Figure 5C–E), suggesting a more efficient ROS detoxification system that protects plants from scald‐induced oxidative stress (Sun, Qin, et al. 2022; Najafi et al. 2024; Sadak, Dawood, and El‐Awadi 2024).

These results are partially consistent with previous studies showing that resistant plants often exhibit stronger antioxidant responses and lower oxidative damage during pathogen infection (Khaledi et al. 2016; Boyno et al. 2022; Najafi et al. 2024). Furthermore, antioxidant genes including *HvCDS1*, *HvSOD2* and *HvCAT1* were significantly upregulated in Yangsimai 3, reflecting its enhanced capacity to mitigate pathogen‐induced oxidative stress (Figure 6A–C). Conversely, expression of *HvRBOHF*, which encodes NADPH oxidases responsible for ROS production, was lower in Yangsimai 3 (Figure 6D), suggesting tighter regulation of ROS generation during infection. Similar transcriptional regulation of antioxidant genes has been reported in soybean during resistance to *Rhizoctonia solani* infection (Samsatly et al. 2018). The lower H_2_O_2_ and MDA levels together with higher SOD, POD and CAT activities in Yangsimai 3 indicate that this cultivar maintained a stronger antioxidant buffering capacity under scald infection. Nevertheless, because antioxidant activity was not experimentally manipulated, these data do not establish that ROS scavenging directly causes resistance. Rather, they identify antioxidant capacity as a physiological trait associated with the resistant response of Yangsimai 3.

### 4.4 Ca^2+^ and Phytohormone Signalling Transcript Was Involved in Scald Resistance in Yangsimai 3

Ca^2+^ signalling and phytohormone‐mediated pathways play central roles in coordinating defence responses following pathogen perception (Zhang et al. 2014; Ding et al. 2022). Ca^2+^ signalling is widely recognized as one of the earliest events during plant pathogen interactions. Pathogen‐triggered Ca^2+^ influx into the cytosol functions as an important secondary messenger that activates downstream defence signalling pathways (Zhang et al. 2014; Ding et al. 2022; Wang and Luan 2024). For example, Lecourieux et al. (2002) demonstrated that treatment of *Nicotiana plumbaginifolia* with the elicitor cryptogein stimulated Ca^2+^ influx, which subsequently triggered defence gene expression, MAPK activation and hypersensitive cell death. Similarly, dynamic cytosolic Ca^2+^ fluctuations have been reported during effector‐triggered immunity (ETI) in the interaction between *Arabidopsis* and *Pseudomonas syringae* (Grant et al. 2000). Consistently, our results showed that the expression of Ca^2+^ signalling genes *HvCAM1*, *HvCAM24* and *HvCDPK3* was significantly upregulated in Yangsimai 3 following scald inoculation compared with Atlantis (Figure 6E–G). The stronger induction of Ca^2+^ signalling, phytohormone‐related, stomatal regulation and MAPK‐associated genes in Yangsimai 3 suggests a broader transcriptional response to *R. secalis* inoculation. However, these expression patterns were only measured at a single time point and do not demonstrate functional roles of these genes in resistance. Therefore, these genes should be considered candidate markers or components associated with contrasting scald responses, requiring further validation through time‐course expression analysis, mutant studies, or gene‐editing approaches.

Phytohormones also play critical roles in regulating plant immune responses against both abiotic stress and pathogen invasions (Sadak, El‐lethy, and Hanafy Amin 2024; Sharmita et al. 2026). In particular, SA and JA signalling pathways are well known to mediate defence responses against a wide range of phytopathogens (Duan et al. 2014; Liu et al. 2016; Qin et al. 2021). In the present study, JA biosynthesis genes *HvAOC*, *HvLOX2* and *HvJIP60*, as well as the SA biosynthesis gene *HvICS*, were significantly upregulated in Yangsimai 3 following *R. secalis* infection compared with Atlantis (Figure 6J–M). These transcriptional responses suggest the activation of a robust hormone‐mediated defence signalling network that contributes to restricting scald infection. Similar hormone‐mediated defence responses have been reported in other plant‐pathogen systems. For example, JA signalling contributes to resistance against *Trichoderma longibrachiatum* infection in cucumber (Yuan et al. 2019). Likewise, SA‐mediated activation of defence‐related genes such as *PR‐5*, *NPR1*, *PR‐2a* and PAL has been shown to enhance resistance against Fusarium wilt in tomato and watermelon (Zhu et al. 2022; Chakraborty 2021).

Overall, Yangsimai 3 exhibits relatively stronger transcriptional activations of genes associated with ion signalling, ROS detoxification and stomatal regulations. These coordinated responses likely contribute to its enhanced resistance against scald infection compared with the susceptible cultivar Atlantis.

## 5 Limitations and Future Directions

First, Atlantis and Yangsimai 3 are genetically distinct cultivars rather than near‐isogenic lines; therefore, the observed differences may reflect both scald resistance‐associated responses and broader genetic background variation. Second, the physiological, biochemical and transcriptional traits measured here were not experimentally manipulated. As a result, these traits should be interpreted as being associated with contrasting scald responses rather than as proven causal determinants of resistance. Third, qRT‐PCR analysis was conducted at a single sampling time point after inoculation, which may not capture the full temporal dynamics of defence signalling. Future studies using NILs, segregating populations, additional resistant and susceptible genotypes, pathogen penetration assays, time‐course transcript profiling and functional validation approaches will be required to confirm the causal roles of stomatal regulation, ion homeostasis, ROS metabolism and defence‐related genes in barley scald resistance.

## 6 Conclusion

This study identified several physiological, biochemical and transcriptional traits associated with contrasting scald responses in barley. Compared with the susceptible cultivar Atlantis, the resistant cultivar Yangsimai 3 maintained more stable stomatal traits, K^+^ and Ca^2+^ contents, lower oxidative damage and stronger antioxidant enzyme activities under scald infection. Yangsimai 3 also showed stronger expression of selected genes associated with Ca^2+^ signalling, ion transport, phytohormone pathways, ROS detoxification and stomatal regulation, providing valuable physiological and molecular insights for breeding scald resistant barley cultivars.

## Author Contributions

Conceptualisation, C.Z. and M.Z.; writing – original draft preparation, U.I.; writing – review and editing, M.A.A. and S.S.; funding acquisition, C.Z.

## Funding

This work was supported by Grains Research and Development Corporation.

## Conflicts of Interest

The authors declare no conflicts of interest.

## Supporting information


**Table S1:** List of primers and their function used in the present study.

**Table S2:** Significance *p*‐values for cultivar effect, treatment effect and cultivar × treatment interaction obtained from two‐way ANOVA.

## Data Availability

The data are available from the corresponding authors upon reasonable request.
